# Long-Term Supplementation with Beta Serum Concentrate (BSC), a Complex of Milk Lipids, during Post-Natal Brain Development Improves Memory in Rats

**DOI:** 10.3390/nu7064526

**Published:** 2015-06-05

**Authors:** Jian Guan, Alastair MacGibbon, Bertram Fong, Rong Zhang, Karen Liu, Angela Rowan, Paul McJarrow

**Affiliations:** 1 Liggins Institute, University of Auckland, 85 Park Road, Grafton, Auckland 1142 , New Zealand; E-Mails: r.zhang@auckland.ac.nz (R.Z.); karen.liu@auckland.ac.nz (K.L.); 2 Centre for Brain Research, the Faculty of Medicine and Health Sciences, University of Auckland, 85 Park Road, Grafton, Auckland 1142, New Zealand; 3 Fonterra Research and Development Centre, Private Bag 11029, Palmerston North 4442, New Zealand; E-Mails: alastair.macgibbon@fonterra.com (A.M.); bertram.fong@fonterra.com (B.F.); paul.mcjarrow@fonterra.com (P.M.); 4 Fonterra Co-operative Group Ltd., Palmerston North 4442, New Zealand; E-Mail: angela.rowan@fonterra.com

**Keywords:** beta serum concentrate, milk fat globule membrane, gangliosides, phospholipids, brain development, memory, Morris water maze, neuroplasticity

## Abstract

We have previously reported that the supplementation of ganglioside-enriched complex-milk-lipids improves cognitive function and that a phospholipid-enriched complex-milk-lipid prevents age-related cognitive decline in rats. This current study evaluated the effects of post-natal supplementation of ganglioside- and phospholipid-enriched complex-milk-lipids beta serum concentrate (BSC) on cognitive function in young rats. The diet of male rats was supplemented with either gels formulated BSC (*n* = 16) or blank gels (*n* = 16) from post-natal day 10 to day 70. Memory and anxiety-like behaviors were evaluated using the Morris water maze, dark–light boxes, and elevated plus maze tests. Neuroplasticity and white matter were measured using immunohistochemical staining. The overall performance in seven-day acquisition trials was similar between the groups. Compared with the control group, BSC supplementation reduced the latency to the platform during day one of the acquisition tests. Supplementation improved memory by showing reduced latency and improved path efficiency to the platform quadrant, and smaller initial heading error from the platform zone. Supplemented rats showed an increase in striatal dopamine terminals and hippocampal glutamate receptors. Thus BSC supplementation during post-natal brain development improved learning and memory, independent from anxiety. The moderately enhanced neuroplasticity in dopamine and glutamate may be biological changes underlying the improved cognitive function.

## 1. Introduction

The first two years of life is a critical period of human brain development that is dominated by rapid neural growth and remodeling [1], particularly neuronal and synaptic plasticity [1], axon development and myelination [2] and vascular remodeling [3]. Although breast milk provides the best nutrition for brain development in the first six months, there is a global need for the development of infant formulas that provide essential and optimal nutrients for brain development in infants with mothers who are unable to breast feed [4].

Gangliosides are sialic-acid-containing glycosphingolipids that are most abundant in the nervous system [5]. The roles of gangliosides in brain development and brain function are well documented [6]. Dairy complex lipids derived from the milk fat globule membrane contain a high concentration of gangliosides, particularly GD3 [7].

The milk fat globule membrane is also rich in phospholipids [8], which are critical components for brain development [9], including white matter formation. The administration of phosphatidylcholine has been shown to improve neuroplasticity and cognitive function [10]. A similar finding has been reported following the administration of Phosphatidylserine (PS) [11]. The combined administration of gangliosides and phospholipids shows promise for promoting the learning ability of children and adolescents [12].

We have previously shown that the long-term oral administration of ganglioside-rich complex milk lipids (CML) during brain development improves novelty recognition and spatial reference learning in a neonatal rat model [13]. Improved synaptic plasticity in the hippocampus has recently been reported to be associated with better memory after the administration of CML [14]. More recently, we have also found that the oral administration of a mixture of dairy phospholipids (complex lipids concentrate) to middle-aged rats prevented the age-related memory decline [15], but not the isolated dairy PS (unpublished data). The result raises the question for the effectiveness of administering “whole food” mixtures *versus* single components.

Cognitive function has broadly been used to evaluate brain function associated with post-natal brain development [13,14]. The hippocampus and the striatum are the brain regions that are involved in cognitive development, including memory formation [16]. Improved neuroplasticity in these brain regions, for example glutamate trafficking in the hippocampus and dopamine neuroplasticity in the striatum, has previously been indicated to be associated with better brain development and enhanced cognitive function [14,15].

Therefore, our hypothesis is that the administration of dairy phospholipids and gangliosides may benefit brain development and cognitive function. Current research evaluated the effectiveness of post-natal administration using the cream-derived complex lipid ingredient beta serum concentrate (BSC), during brain development by measuring later cognitive function and its associated neuroplasticity and white matter development.

## 2. Methods

### 2.1. Animal Experiments

The animal experiments were approved by the Animal Ethics Committee, the University of Auckland (001260). The litter size was normalized at birth to eight pups per litter. Eight litters of rats (Wistar, dam plus four male pups per litter) were received from the animal resource unit of the University of Auckland at post-natal (PN) day 7. Each dam and her pups were hosted in a standard cage with *ad libitum* access to a standard diet (Diet 2018; Harlan Teklad, Oxon, UK) [13] and water in the room with 12:12 h light/dark cycle. At PN day 10, which is similar to human term new borns the 32 pups were randomly allocated to two supplementation groups of either blank gelatin (*n* = 16) or gelatin formulated with cream-derived BSC (*n* = 16) supplied by Fonterra Co-operative Group Limited) for the next 60 days. The pups were weaned at PN day 21. Body weight was measured every 3 days throughout the experiments. The behavioral tests were conducted from PN day 40 to PN day 69, for which the stages of brain development of rats are similar to that of pre- to early adolescence human brains. The experiments were terminated at PN day 70. The brain tissues were collected at the end of the experiment for biological analysis and the body composition of the carcasses was measured after tissue collection.

### 2.2. Gelatin Formulation and Supplementation

Gels were formulated using raspberry flavoring and gelatin (10% w/v) containing sucrose (10% w/v) [15]. The BSC supplement was mixed in water using a food processor and was then mixed with 1 L of the flavoring/gelatin/sucrose mixture at 50 °C. The mixture was then transferred into ice cube trays and firmed at 4 °C. Each cube contained 12.5 mL of gelatin with or without BSC.

The composition of the BSC is described in Table 1. Rats were hand fed from PN day 10 to PN day 21 (weaning day) and were individually cage fed thereafter until PN day 69. The dose of BSC was 5.0 mg/g/day, which was calculated daily based on the body weight of the individual rat. The control group was fed with blank gelatin (BG), *i.e.*, prepared without the supplement. The rats were returned to their home cage after they had consumed the gels.

**Table 1:** Composition of beta serum concentrate (BSC) (g/100 g).

Protein	52.30
Total fat	36.20
Phospholipids	13.67
Gangliosides (GD3)	0.63
Lactose	6.60
Minerals	5.20

There was less than 1% of GM3 of total gangliosides.

### 2.3. Behavioral Tests

The behavioral tests were carried out in the following order: the dark–light boxes tests performed on PN day 40, Morris water maze (MWM) tests, which were conducted between PN days 55 and 65, and a 1-day elevated plus maze (EPM) test performed on PN day 69. The activity during the tests was recorded and analyzed with automated tracking software (ANY-maze, v4.2, Stoelting Co., Wood Dale, IL, USA).

### 2.4. Dark–Light Boxes Tests

The test arena is an open-top square transparent plastic box (60 cm × 60 cm × 60 cm). Half of the box was occupied with a black box that has a lid on the top and a small commuting door that is open to the light part of the box. During the test, a rat was put into the dark box through the lid and was allowed to explore both boxes for 5 min. The time spent in, and the number of entries to, the light box was recorded and were analyzed using the ANY-maze tracking system. The definition of entry was 80% of the body in the light box.

### 2.5. Morris Water Maze (MWM) Tests

#### 2.5.1. Apparatus

The MWM tests were carried out in a quiet room with multiple distal cues around the testing apparatus (e.g., a bright yellow triangle and a large red cross). A black circular pool (0.6 m × 2.2 m in diameter) was filled with water (maintained at 20–22 °C) and a submerged transparent plastic platform (10 cm in diameter) was located in the southwest quadrant 20 cm away from the side wall.

#### 2.5.2. Acquisition Testing

The procedure has been described previously [15]. In brief, rats were given four trials on each of 7 training days, with 6-min intervals between trials. The starting position of the trial was randomized over each training day and each trial during the testing day, but remained the same for all the rats. The platform remained in the SW quadrant throughout the tests. During each trial, the rat was placed in the water with its head pointed towards the side wall and was allowed to swim for 120 s to locate and mount the hidden platform. To enforce the spatial learning, the rat was left on the platform for 15 s before being removed after each trial. The latency, total distance travelled, and swimming speed taken to reach the platform were recorded and analyzed. Rats that were unable to locate the platform within 120 s were guided to the platform and remained on the platform for 15 s to learn the location of the platform.

#### 2.5.3. Test Trial

Three test trials were conducted to examine spatial reference memory at 24, 48, and 72 h after the last acquisition trial. During the test trials, the platform was removed from the pool and the rat was allowed to spend 30 s in the pool searching for the absent platform before being removed from the pool. The initial heading error to the platform zone, the number of entries to the platform quadrant, and the path efficiency to the platform quadrant were analyzed for the retention of spatial memory.

### 2.6. Elevated Plus Maze (EPM) Test

The procedure for measuring anxiety-like behavior with the elevated maze has been described previously [15,16]. The apparatus consists of two open arms (flat platform, 50 cm × 10 cm) and two enclosed arms (flat platform enclosed by walls, 50 cm × 10 cm × 40 cm) perpendicularly intersecting to form a central platform. The maze was elevated 50 cm above the floor. During the test, the rat was placed on the central platform, facing an open arm, and was allowed to explore the maze freely for 5 min. The definition of an arm entry is when 80% of the body was inside the arm [17]. The number of entries to and the time spent in the open arms were recorded and were used as a measure of anxiety-like behavior.

### 2.7. Tissue Collections

Rats were deeply anesthetized with pentobarbital (125 mg/kg, i.p.) and blood samples (1–2 mL) were then collected via cardiac punctures. The rats were transcardiacally perfused with normal saline until the outflow from the heart ran clear. The brains were collected and separated into two hemispheres. The left hemisphere was frozen in dry ice immediately and was stored at −80 °C. The right hemisphere was fixed *in situ* with 4% paraformaldehyde for histological analysis. Bone density and body composition were examined using dual energy X-ray absorptiometry scanning after tissue collection.

### 2.8. Immunohistochemical Staining

The section collection and the preparation for immunohistochemical staining have been reported [15]. Briefly, sequential sections (coronal, 25 µm) were collected, with every 12 sections pooled together (e.g., sections 1, 13, 25, 37, and so on). The sections from each sample pool were used for one parameter of staining, which was performed simultaneously across the supplementation groups.

Primary antibodies against tyrosine hydroxylase (TH), synaptophysin, glutamate receptor 1 (GluR-1), choline acetyltransferase (ChAT), and myelin basic protein (MBP) were used to mark, respectively, dopamine terminals, the sub-unit of α-amino-3-hydroxy-5-methyl-4-isoxazolepropionic acid (AMPA) receptors, cholinergic interneurons, and the myelin sheath in the striatum and the hippocampus. The sections were pretreated with 1% H_2_O_2_ in 50% methanol for 30 min to quench endogenous peroxidase activity, and then were incubated with 1.5% normal sheep serum/phosphate-buffered saline at room temperature to block non-specific staining. The sections were then incubated with the following primary antibodies: rabbit anti-TH antibody (Protos Biotech Corporation, 1:1000), rabbit anti-GluR-1 (Chemicon, 1:10,000), mouse anti-MBP (Jomar Millipore 1:10000), and goat anti-ChAT (Santa-Cruz 1:2000), at 4 °C for 48 h. Sections were incubated with either biotinylated horse anti-mouse or goat anti-rabbit secondary antibodies accordingly (1:2000, Sigma) at 4 °C overnight. ExtrAvidin (Sigma, 1:2000) was applied for 3 h at room temperature and 0.05% 3,3-diaminobenzidine was then added to produce a brown reaction product. The sections were washed (10 min × 3) between the incubation periods. The stained sections were then mounted on gelatin-coated slides, dehydrated in a series of alcohols to xylene, and cover slipped with the mounting medium.

### 2.9. Data Assessments

Photographs of the hippocampus and the striatum were taken using a microscope (Nikon 800i, Tokyo, Japan) and a digital camera system (Nikon Digital Sight, Tokyo, Japan). Four images were taken from each brain region in at least six sequential sections. The average densities of TH, GluR-1, and MBP staining were measured in the striatum and the sub-regions of the hippocampus, respectively, using image analysis software (Image J and SigmaScan Pro 5.0, SPSS, Chicago, IL, USA) and the light microscope at 4× magnification. The number of ChAT-positive neurons was counted manually in the striatum. The area used for counting cells was measured and was used for converting the total number to density as cells/mm^2^. The personnel involved in the data assessments and analyses were blinded to the experimental groups.

### 2.10. Data Analysis

The data generated from the MWM tests, body weights, and the expression of GluR-1 and synaptophysin were analyzed using two-way analysis of variance (ANOVA) for comparing the differences between the groups and time points/brain regions respectively (Graphpad Prism version 6.01). The data from body composition, the EPM test, and TH density were analyzed using two-tailed *t*-tests. A *p*-value of ≤0.05 was considered to be significant. The data are presented as means ± standard error of the mean (SEM).

## 3. Results

### 3.1. Body Weight and Body Composition

**Figure 1:**
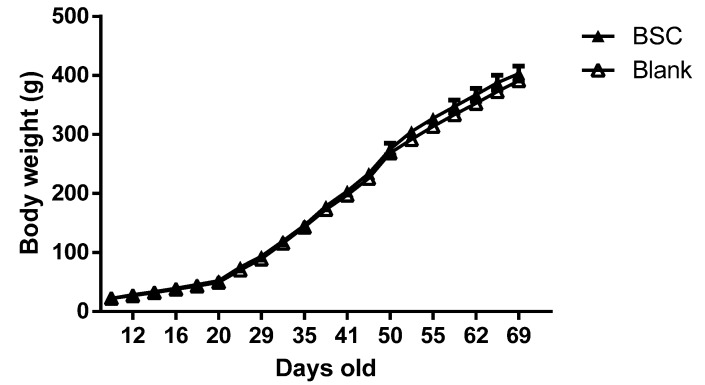
Effects of BSC supplementation with beta serum concentrate (BSC) on body weight;.two-way ANOVA analysis. The data are presented as mean ± SEM, *n* = 16.

The two-way ANOVA analysis suggested that BSC supplementation did not alter the body weight compared with the control group throughout the experimental period (Figure 1).

Two-tailed *t*-tests did not suggest any differences between the groups in the ratio of lean/fat mass percentage of body fat, the average bone mineral density and the body length (Table 2).

**Table 2:** Body composition of the rats.

	BG	BSC
Fat mass (%)	25 ± 1.12	25 ± 1.08
Fat/lean ratio	0.34 ± 0.02	0.34 ± 0.01
Bone mineral density (g)	7.5 ± 0.22	7.3 ± 0.18
Body length (cm)	40.17 ± 0.43	40.71 ± 0.52

BG: blank gel, control group; BSC: beta serum concentrate group.

### 3.2. Plasma Lipid Composition

The lipid composition of the plasma was analyzed using an auto analyzer (Roche Hitachi, 902 Automatic Analyzer). The supplementation did not alter the concentrations of triglyceride, cholesterol, low-density lipoprotein cholesterol, and high-density lipoprotein cholesterol compared with the control group (Table 3).

**Table 3:** Concentrations of plasma lipids.

Plasma (mmol/L)	BG	BSC
Triglyceride	1.46 ± 0.08	1.33 ± 0.11
Low density lipoprotein cholesterol	0.33 ± 0.02	0.35 ± 0.02
High density lipoprotein cholesterol	1.51 ± 0.2	1.40 ± 0.4
Cholesterol	2.07 ± 0.08	2.07 ± 0.05

BG: blank gel, control group; BSC: beta serum concentrate group.

### 3.3. Dark–Light Box Tests

**Figure 2:**
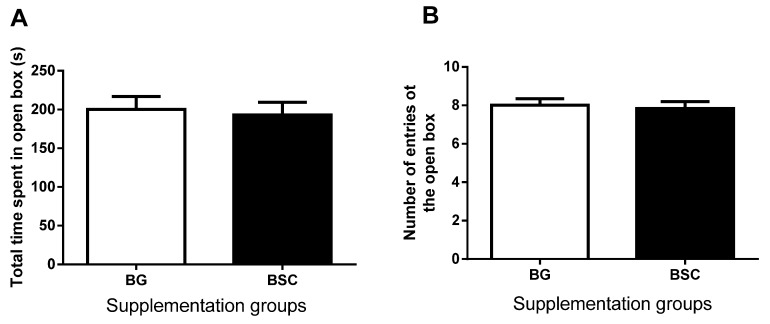
Performances in the dark–light boxes tests. (**A**) The time spent in and (**B**) the number entries to the light box with (closed bars, BSC) and without (open bars, BG) the supplementation. The data are presented as mean ± SEM, *n* = 16.

The one-day test was conducted on PN day 40. The time spent in (Figure 2A), and the number of entries to (Figure 2B) the light box, were similar between the two groups.

### 3.4. Morris Water Maze Tests

#### 3.4.1. Acquisitions

**Figure 3:**
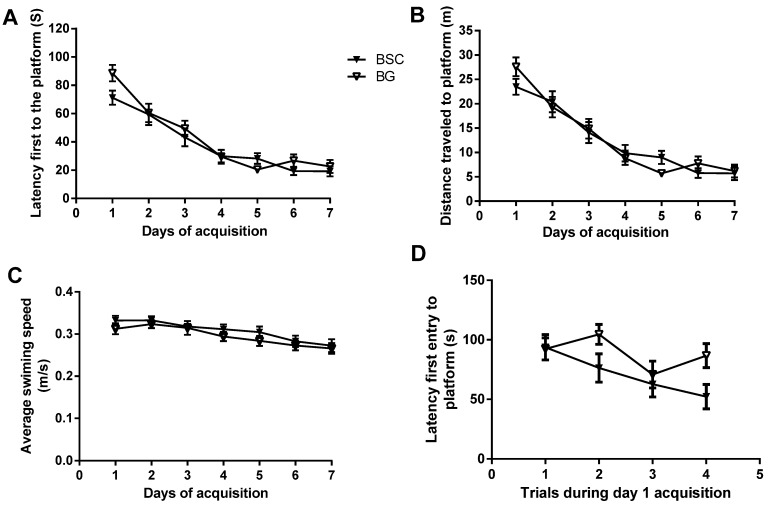
Performances in the Morris water maze tests performance during 7 days of acquisition trials, which were evaluated by analyzing (**A**) the latency, and (**B**) the distance travelled to the platform, and (**C**) the swimming speed and (**D**, *p* < 0.05) day 1 acquisition trials. The data are presented as mean ± SEM, *n* = 16, BG = blank gel.

The acquisition tests were conducted on PN days 55–62. Figure 3 shows (A) the time taken to find the platform (latency), (B) the distance traveled to locate the platform, and (C) the swimming speed during the 7 days of acquisition tests. Two-way ANOVA revealed a significant reduction in the latency (Figure 3A, *p* < 0.0001, F (6,210) = 39.93, *n* = 16) and the distance traveled to locate the platform (Figure 3B, *p* < 0.0001, F (6,210) = 41.71, *n* = 16) over the 7 days of training. There was no difference between the groups and no interactions between the groups and the day of training. There was no difference in swimming speed between the groups and the training days (Figure 3C).

Given the potential difference in day 1 acquisitions between the groups, we also analyzed the latency of the four tests during the day 1 acquisition trial. The latency of the first trials was identical between the two groups. In addition, a two-way ANOVA showed a significant difference in latency first to the platform between the groups (Figure 3D, *p* = 0.02, F (1,120) = 5.53) and within the trials (*p* = 0.01, F (1,120) = 3.12); there was no interaction between the time points and the group effects. The data suggested that the rats with BSC supplementation learned to locate the platform better than the control rats during the first training day.

#### 3.4.2. Test Trials

**Figure 4:**
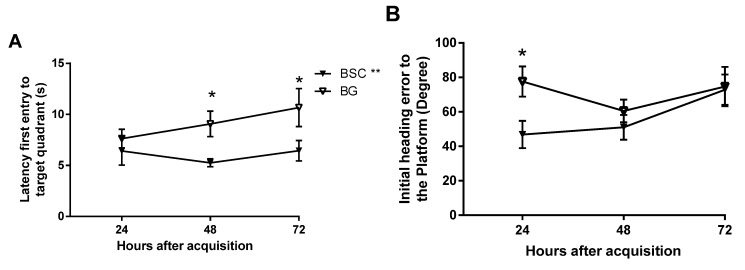

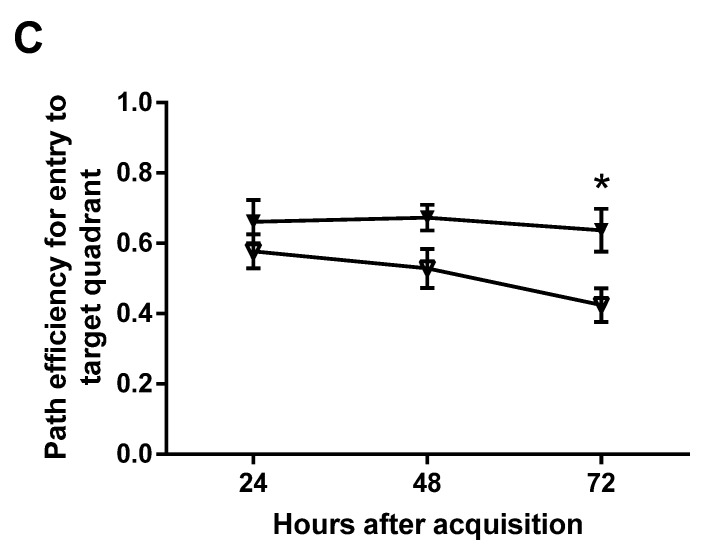
MWM performance in testing trials evaluated at 24, 48, and 72 h after the acquisition tests. The memory retention was analyzed using the latency to (**A**) and the path efficacy to the targeted quadrant (**B**) the targeted quadrant and the initial heading error from the platform zoon (**C**). The data are presented as mean ± SEM, *n* = 16, BG = blank gel, * *p* < 0.05, ** *p* < 0.01.

The test trials were conducted during PN day 63–65. Memory retention was evaluated at 24, 48, and 72 h after the last acquisition trial. Figure 4 shows (A) the latency of first entry, (B) the initial heading error towards the platform zone, and (C) the path efficiency to the target quadrant. Two-way ANOVA showed that the latency of first entry to the target quadrant was significantly reduced in the BSC group compared with the control group (Figure 4A, *p* = 0.0027, F (1,87) = 9.5). There was no difference between the time points and no interactions between the groups and the time points. The multiple comparisons suggested that the latency was significantly reduced at both 48 and 72 h (Figure 4A, *p* < 0.05).

Two-way ANOVA also revealed that the initial heading error from the platform was significantly reduced in the BSC group compared with the control group (Figure 4B, *p* = 0.048, F (1,90) = 3.98, *n* = 16) and that there was no difference between the time points and no interactions between the groups and the time points. Post-hoc tests suggested a significant reduction in the initial heading error at 24 h after the last acquisition trial (Figure 4B, *p* < 0.05).

The path efficiency to the platform quadrant was significantly improved in the BSC group compared with the control group (Figure 4C, *p* = 0.0009, F (1,87) = 11.76). There was no difference between the time points and no interaction between the groups and the time points. Multiple comparisons indicated a significant increase in the path efficiency at 72 h after the last acquisition trial (Figure 4C, *p* < 0.05).

### 3.5. Elevated Plus Maze Tests

**Figure 5:**
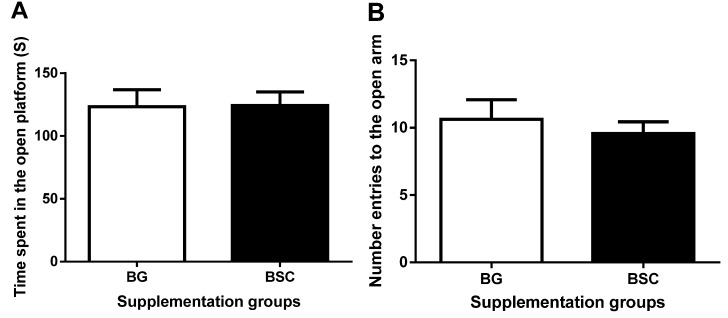
The time spent in (**A**) and the number of entries to (**B**) the open arms of Elevated Plus Maze were similar between the groups with (closed bars) and without (open bars) the supplementation. The data are presented as mean ± SEM, *n* = 16, BG = blank gel.

EPM tests were conducted in PN day 69. The time spent in (Figure 5A), and the number of entries to the open platforms (Figure 5B), were similar between the two groups. The data suggested that the anxiety-like behavior after the MWM tests was similar between the two groups.

### 3.6. Immunohistochemistry

**Figure 6:**
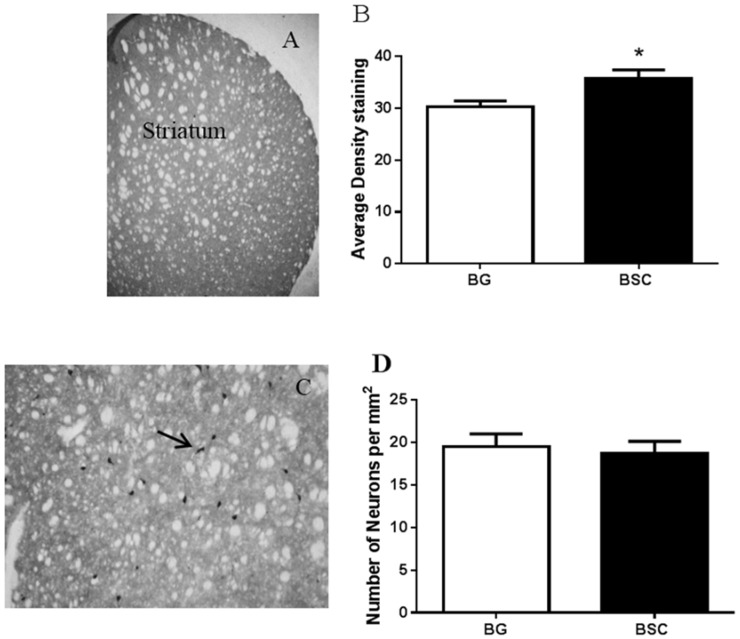

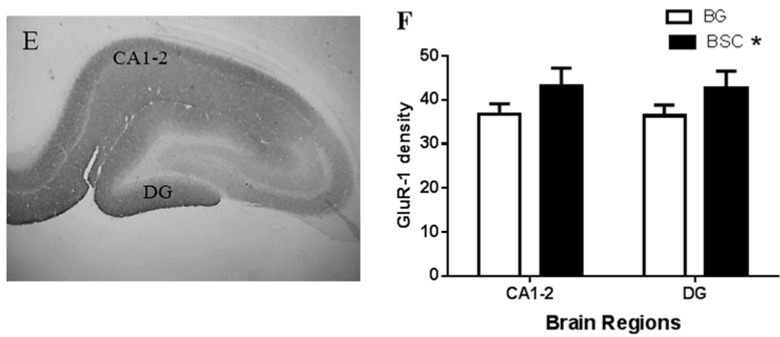
Analysis of the TH density (TH, **A**,**B**) and the ChAT -positive neurons (**C**,**D**) in the striatum and the glutamate receptor-1 density in the hippocampus (**E**,**F**). The data are presented as mean ± SEM, *n* = 16, BG = blank gel, * *p* < 0.05.

Figure 6 shows the changes in TH density (Figure 6A,B) and ChAT-positive neurons in the striatum (Figure 6C,D) and the GluR-1 density in the CA1-2 sub-regions of the hippocampus and the dentate gyrus (Figure 6E,F).

The TH staining in the striatum was located at the terminal of dopamine neurons projected from the substantia nigra. The terminal staining was evenly distributed throughout the striatum (Figure 6A). The average density of TH staining was significantly increased in the striatum of the rats with BSC supplementation compared with the control rats (Figure 6B, *p* = 0.0027, *n* = 16).

The ChAT-positive staining was expressed in the cholinergic interneurons, which were distributed within the striatum (Figure 6C). There was no difference between the two groups in the number of ChAT-positive neurons in the striatum (Figure 6D).

The staining of GluR-1 was dense and clearly located in the oriens layer and the stratum radiatum of the CA1-2 sub-regions of the hippocampus and the dentate gyrus (Figure 6E), in which the average density of GluR-1 was assessed. The staining of GluR-1 in the CA3 and CA4 sub-regions of the hippocampus was mainly neuronal and relatively weaker (data not shown). Two-way ANOVA suggested a moderate increase in the density of GluR-1 staining with BSC supplementation (*p* = 0.05, Figure 6F). Multiple comparisons did not suggest any difference in specific brain regions. The average density of MBP staining was similar between the groups and in all brain regions analyzed.

## 4. Discussion

In general, long-term supplementation with BSC gangliosides and phospholipids to young rats during post-natal brain development improved spatial reference learning and memory independent of anxiety-like behavior. The promotion of dopamine and glutamate neuroplasticity may underlie the improved brain function.

The first two years of life are critical for brain development, which continues into adolescence [4]. PN day 10 to PN day 70 in rats is developmentally similar to that of the human brain from newborn to early adolescence, as the brain matures at around three months of age in Wistar rats. Even though supplementation during the period of brain development did not improve the ability of learning during the seven-day acquisition training, there was a separation of latency between the two groups during day 1 acquisition (Figure 3A). To examine whether the starting point of acquisition was similar between the two groups, we compared the latencies of four trials during day 1 acquisition tests. The analysis showed that the latency of the first trial was identical between the two groups. In addition, the analysis also suggested an overall reduction in latency in the rats with supplementation. The supplementation with BSC may have moderately improved spatial reference learning (Figure 3D). The rats used for the study were normal with no obvious deficits. Unlike a study testing the effects of pharmaceutical interventions in animal models with behavioral deficits, the efficacy of a nutritional supplementation in normal rats is likely to be mild and thus difficult to detect. It usually takes a minimum of two days for normal rats to learn the task of locating the hidden platform. The analysis of the day 1 acquisition trial may provide an opportunity to detect the mild and transient efficacy, even though the effects may have disappeared following subsequent training.

Similarly, the delayed tests on memory retention may also help to identify the subtle differences between the groups [15]. The test trials are usually conducted two hours after the completion of acquisition training for short-term memory and 24 h afterwards for delayed memory. As we aimed to detect mild improvement and whether the memory is long lasting, we added two later time points by testing the memory at 24, 48, and 72 h after training. The design would also provide the changes in memory over a period of three days. Memory was analyzed in several parameters and three showed improvement, some at later time points and others at earlier time points of the memory tests. Supplementation improved memory by showing reduced latency to, and improved path efficiency to, the target quadrant, as well as reduced initial heading errors from the platform. Although there was no difference at 24 h, we detected a significant improvement in both latency and path efficiency to the targeted quadrant at later time points (Figure 5A,C) because of well-maintained effects on memory retention. The rats with supplementation made smaller initial heading errors at the 24-h time point, but failed to maintain the advantage. The parameters that detected the memory improvement at later time points may be the sensitive measures for mild effects, which would be missed if the memory tests were conducted within 24 h following the acquisition.

It has been suggested that the phospholipid-associated improvement in memory may be mediated through reduced anxiety [18]. We have recently reported improved cognitive function after the supplementation of two different CML preparations [13,15]. Better cognitive function is clearly associated with improved neuroplasticity of dopamine [14,15], a neurotransmitter that is involved in anxiety [19]. As a forced escape behavior, the tasks of the MWM test may cause anxiety. To evaluate whether the improved performance in the MWM test may be related to anxiety, we carried out anxiety tests before and after the MWM tests. The anxiety-like behavior evaluated by both dark–light boxes tests and EPM tests was similar between the groups, suggesting that the improved memory after supplementation was not due to the reduction in anxiety.

Apart from anxiety [19,20], dopamine neurotransmission plays a critical role in cognition [21]. By evaluating the expression of TH, the enzyme that synthesizes dopamine, we found a moderate, but significant, increase in dopamine output in the striatum of rats with supplementation compared with the control rats. Promoting dopamine output has consistently been associated with memory improvement as a result of CML supplementation [14,15]. Thus, the promotion of dopamine neurotransmission may be the key biological change underlying the improved ability in learning and memory, rather than an indirect effect of reduced anxiety, because the cholinergic interneurons in the striatum receive some of the dopamine projections [22] and cholinergic neurotransmission may involve BSC-associated memory improvement [16]. However, the results suggested that the improved cognitive function was not associated with the expression of ChAT, the enzyme that synthesizes acetylcholine, despite a moderate increase in dopamine output (Figure 6A,B).

In addition, we observed a mild increase in GluR-1 density in the CA1-2 sub-region of the hippocampus and the dentate gyrus (Figure 6F). GluR-1 is a sub-unit of the AMPA receptor, a sub-type of glutamate receptors, and the AMPA receptor trafficking in the hippocampus is critical in memory and learning [23]. It has been reported that the activation of AMPA receptors increases the expression of several isoforms of gangliosides in the hippocampus [24]. Gangliosides have the ability to regulate the function of the AMPA receptor [25]. Similarly, the association between phospholipids and glutamate trafficking has also been reported, for example the ability of phosphatidylinositol in maintaining glutamate synaptic trafficking [26].

Improved cognitive function due to gangliosides [5,27] and phospholipids [28] could also be mediated through promoting myelination. However the supplementation of BSC did not alter the myelin density in all the brain regions examined. The lack of effect on myelination has recently been reported following the supplementation of ganglioside-enriched CML in a similar supplementary regime [14].

## 5. Conclusions

In conclusion, supplementation with a mixture of milk-fat-globule-membrane-derived BSC during the critical period of brain development improved learning and memory in young adult rats. The improved cognitive function was not associated with anxiety-like behavior. The supplementation also improved dopamine and glutamate neurotransmission, which may be the neurobiological mechanisms underlying the improved cognitive function.
